# Establishment of the cytoplasmic incompatibility-inducing *Wolbachia* strain *w*Mel in an important agricultural pest insect

**DOI:** 10.1038/srep39200

**Published:** 2016-12-16

**Authors:** Xiao-Fei Zhou, Zheng-Xi Li

**Affiliations:** 1 Department of Entomology, China Agricultural University, 2 Yuanmingyuan West Road, Beijing 100193, China

## Abstract

The *w*Mel *Wolbachia* strain was known for cytoplasmic incompatibility (CI)-induction and blocking the transmission of dengue. However, it is unknown whether it can establish and induce CI in a non-dipteran host insect. Here we artificially transferred *w*Mel from *Drosophila melanogaster* into the whitefly *Bemisia tabaci*. Fluorescence *in situ* hybridisation demonstrated that *w*Mel had successfully transfected the new host. Reciprocal crossing was conducted with *w*Mel-transfected and wild-type isofemale lines, indicating that *w*Mel could induce a strong CI without imposing significant cost on host fecundity. We then determined the maternal transmission efficiency of *w*Mel in the offspring generations, showing a fluctuating trend over a period of 12 generations. We thus detected the titre of *w*Mel during different developmental stages and in different generations by using real-time quantitative PCR, revealing a similar fluctuating mode, but it was not significantly correlated with the dynamics of transmission efficiency. These results suggest that *w*Mel can be established in *B.tabaci*, a distantly related pest insect of agricultural importance; moreover, it can induce a strong CI phenotype in the recipient host insect, suggesting a potential for its use in biological control of *B. tabaci*.

Maternally inherited microorganisms are common and have diverse effects on invertebrate biology^1^. *Wolbachia* are the most widely studied of these endosymbionts, and 40% of terrestrial arthropod species and approximately 66% of all insect species are infected with *Wolbachia*^2^,^3^. They are master manipulators of arthropod reproduction and induce a number of reproductive abnormalities, including cytoplasmic incompatibility (CI), feminization, male killing and parthenogenesis induction, all of which enhance the spread of *Wolbachia* in host populations^4^,^5^,^6^. Among these, CI is the most commonly reported phenotype caused by *Wolbachia*, which has caught the attention of many researchers for its potential as a useful strategy for biological control of pest insects of medical and agricultural importance^7^,^8^,^9^.

CI occurs when conspecific insects with different *Wolbachia* infection status mate, which causes embryonic mortality in diploid species but results in sex distortion in haplodiploid species, such as in *Bemisia tabaci*, where male offspring is produced from unfertilised eggs^10^,^11^. CI can be either unidirectional or bidirectional. The former occurs when an uninfected female mates with an infected male, while the reciprocal mating is compatible; the latter is expressed between conspecifics infected with different *Wolbachia* strains. *Wolbachia*-induced CI was exploited as a method for pest population suppression in a very early time^12^. The prerequisite for a CI-based pest control strategy is the obtainment of incompatible male insects. In early attempts, incompatible insects were obtained through introgressive hybridisation and released to control disease vectors in a way analogous to the sterile insect technique^13^,^14^. However, this process is time-consuming and laborious. Since then, artificial interspecies transinfection of *Wolbachia* strains has been developed through embryonic microinjection^15^,^16^. To date, experimental transinfection of *Wolbachia* endosymbionts between different host species has been repeatedly achieved through either embryonic or nymphal injection^17^,^18^,^19^,^20^,^21^,^22^,^23^,^24^. Artificial transinfection has greatly facilitated the application of CI-based technologies in pest population suppression^25^,^26^,^27^. The most famous case was the successful establishment of the *w*Mel *Wolbachia* strain from *Drosophila melanogaster* in *Aedes* populations to block dengue transmission due to its effective interference with RNA viruses^28^,^29^,^30^,^31^. The *Wolbachia* strain *w*Mel originating from *D. melanogaster* has spread globally within the last century^32^. Despite the knowledge that *w*Mel could induce CI and invade the dipteran *Aedes* vectors^33^,^34^,^35^, it is still unknown whether it can establish and induce CI in a distantly related host insect.

*B. tabaci* (Hemiptera:Aleyrodidae) is a species complex with a global distribution, which has caused considerable damages to ornamental, vegetable, grain legume and cotton production^36^, particularly the Middle East-Asia Minor 1 (MEAM1 or B biotype) and Mediterranean (MED or Q biotype)^37^. Due to the serious problem of insecticide resistance in this species, alternative control approaches are needed^38^, and *Wolbachia*-induced CI might be a useful strategy since the natural populations of *B. tabaci* (especially the B and Q biotypes) are widely infected with *Wolbachia* and antibiotic treatment could induce strong CI in B biotype^39^,^40^. Nevertheless, before any biological control programs can really be tested, an isofemale line stably infected with a heterologous CI-inducing *Wolbachia* strain should be established. Here we isolated the *w*Mel strain from a local *D. melanogaster* population and then transferred it into *B. tabaci* through nymphal microinjection. After localisation of *w*Mel in the new host, transfected isofemale lines were established and reciprocal crossing experiments were thus conducted. Subsequently, we measured the titre of *w*Mel at different developmental stages of the new host over a total of 12 generations by using real-time quantitative PCR (qPCR), and determined the transmission efficiency of *w*Mel in the offspring population. The aims of this study were to determine (i) whether *w*Mel could establish in a distantly related host insect; (ii) the CI-inducing capability of *w*Mel in a pest insect of agricultural importance; (iii) the dynamics of the titre of *w*Mel, and (iv) the correlation between titre and transmission rate. Our experimental data suggested that *w*Mel could establish in the new host and induce a strong CI; while the titre and transmission rate shared a similar fluctuating mode at different developmental stages and between generations, the transmission rate of *w*Mel was not necessarily determined by its titre.

## Results

### MLST typing, transinfection and establishment of isofemale line

The *Wolbachia* strain isolated from *D. melanogaster* was identified by gene sequencing and MLST typing^41^. A batch sequence query in the PubMLST database revealed that its allelic profile for *gatB, coxA, hcpA, ftsZ* and *fbpA* was 1, 1, 1, 1 and 1 (corresponding to ST-1), and its HVR profile for HVR1, HVR2, HVR3 and HVR4 was 1, 12, 21 and 24 (corresponding to ST-31) (Supplementary Table S1). A comparison of its sequence types with the STs in the database showed that the MLST and HVR profiles of this strain exactly matched those of the strain Dmel_A_*w*Mel from *D. melanogaster* (PubMLST ID: 1), belonging to Supergroup A^42^. Dmel_A_*w*Mel is a typical CI-inducing *Wolbachia* strain. We also examined the sub-strain status of the strain, showing that it is *w*Mel but not *w*MelCS since only the primers^32^ targeting IS5-WD0516/7 could produce amplicon of expected size (~2500 bp).

The purified *Wolbachia (w*Mel) was directly transferred into the 4^th^ instar nymphs (pseudopupae) of *B. tabaci* by microinjection. Approximately 80–100 4th-instar whitefly nymphs (pseudopupae) were microinjected for each batch, and the survival rate was 50–60%. The *w*Mel-transfected nymphs were maintained in a climate incubator until eclosion after approximately 72 h (Supplementary Fig. S1). When the whiteflies emerged (G_0_), they were confirmed for positive infection with *w*Mel strain by using *Wolbachia wsp*-based PCR detection, and infected adults were moved to a nylon mesh cage for establishment of isofemale lines.

### Localisation of *w*Mel

**Figure 1:**
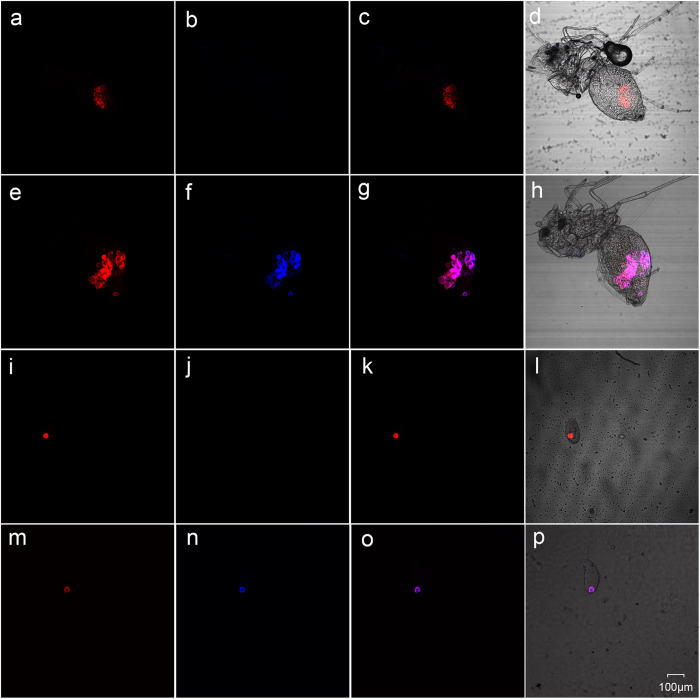
FISH analysis of *w*Mel-transfected and wild-type *B. tabaci* with *Portiera*-specific probe (red) and *Wolbachia*-specific probe (blue). (**a–d**) Wild-type female adult; (**e–h**) Transfected female adult; (**i–l**) Wild-type egg; (**m–p**) Transfected egg. (**a,e,i,m**) *Portiera* channel only; (**b,f,j,n**) *Wolbachia* channel only; (**c,g,k,o**) Merged images showing overlap of *Wolbachia* and *Portiera* channels in dark field; (**d,h,l,p**) Merged images showing overlap of *Wolbachia* and *Portiera* channels in bright field.

FISH was employed to localize the *w*Mel strain in the new host. The results showed that both *Portiera* and *Wolbachia* were localized inside the bacteriocytes; *w*Mel existed along with the primary endosymbiont *Portiera* throughout the life cycle of transfected *B. tabaci* (egg, nymph and adult), though the titres of *w*Mel were different during different developmental stages (Fig. 1). *Wolbachia* gave a specific blue light, while *Portiera* released a strong red signal. The wild-type *B. tabaci* hosted only *Portiera* without *w*Mel being observed. Our FISH data suggested that the *w*Mel strain had been successfully transfected into the recipient host, and also demonstrated that it could be transmitted from mother to offspring through eggs (see Supplementary Fig. S2 for a full set of FISH images).

### Crossing and CI analysis

Totally four different crossing experiments were conducted: (WT♀ × WT♂); (WT♀ × TI♂); (WT♂ × TI♀), and (TI♀ × TI♂). The whiteflies are taken from G_6_. The results showed that there was significant difference in the number of offspring per female between (WT♀ × TI♂) (16.1 ± 0.53) and (TI♀ × TI♂) (19.8 ± 0.97) (SNK, *P* = 0.006), but no significant difference was found between the other crossings. There was an extremely significant difference in the number of male offspring among the four crossings (SNK, *P* = 0.0001), though no significant difference was observed between (WT♀ × WT♂) (53.56 ± 2.49) and (TI♀ × TI♂) (58.35 ± 1.89) (SNK, *P* = 0.074), both of which gave approximately equal mean number of male and female progenies (Table 1). A simple criterion for judging CI level in *B. tabaci* (a haplodiploid species) is to calculate the male/female ratio in the progenies: if CI is induced, the percentage of male offspring will increase as the unfertilised eggs produce male offspring. In the present study, the highest mean percentage of male offspring (97.46%) was observed in the crossing (WT♀ × TI♂), but surprisingly the reciprocal crossing (WT♂ × TI♀) (n = 11) also produced more male (68.13%) than female progenies. We conducted a *Chi*-square test to analyse the male-biased reciprocal crossing result, suggesting that the difference between the observed and expected (1:1) numbers of male offspring is not significant (Pearson *χ*^2^ = 1.029, *df* = 1, *P* = 0.310). Our crossing data indicated that nearly complete CI was induced between TI♂ and WT♀ since the offspring from this crossing was almost all male, but the partial CI in the reciprocal direction (WT♂ × TI♀) is not statistically supported.

### Maternal transmission rate

**Figure 2:**
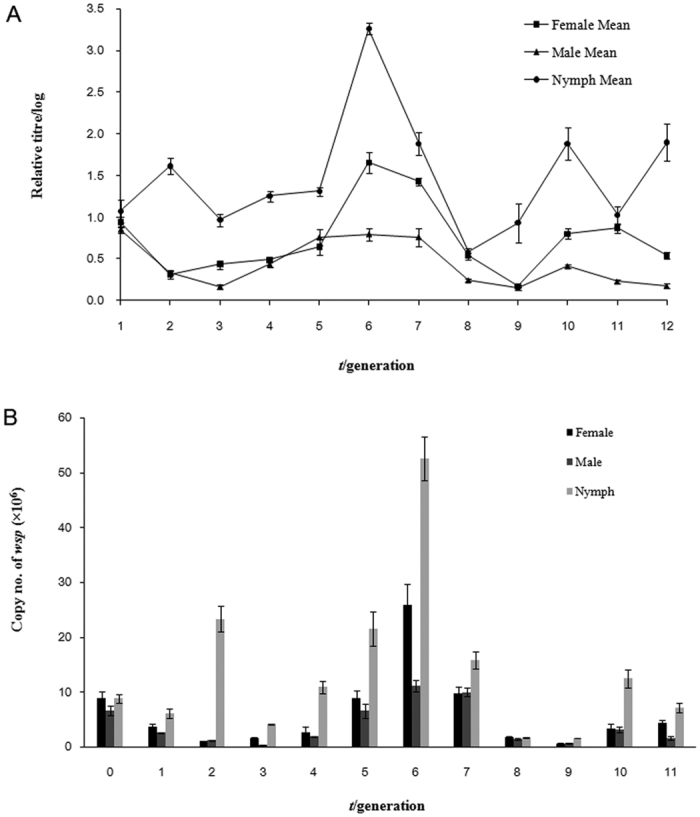
Infection dynamics of the *w*Mel strain in the recipient host *B. tabaci* at different developmental stages and in different generations. (**A**) Log relative titre of *w*Mel in *B. tabaci*. (**B**) The copy number of *w*Mel in *B. tabaci*.

Transmission rate is an important parameter for estimating the transmission efficiency of a *Wolbachia* strain, which determines its potential as a biocontrol agent. Our data showed that the transmission rates of *w*Mel fluctuated between generations: reaching a lowest point at G_2_ (10.72%), then gradually climbing up to a peak point at G_6_ (87.5%) and heading down again, followed by a slight rebound (Fig. 2). It seems that the transmission rate of *w*Mel may fluctuate around the equilibrium level (~50%) in the next generations if more generations are investigated. In a period of 12 generations, the artificially transfected *w*Mel was shown to be transmitted in the offspring populations with a fluctuating mode.

### Dynamics of the titre of *w*Mel

**Figure 3:**
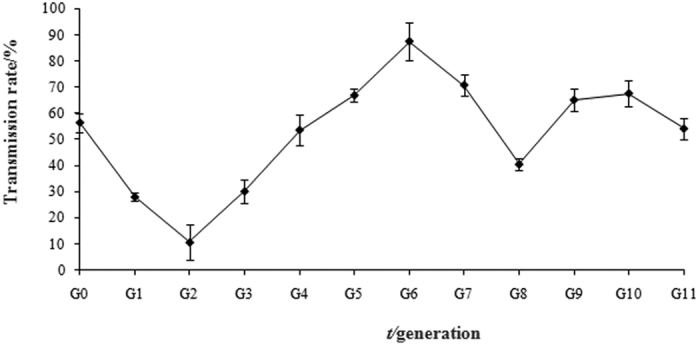
Maternal transmission rate of the *w*Mel strain in offspring generations. Data are mean ± S.E., derived from three independent isofemale lines.

The fluctuating transmission rate of *w*Mel observed above may be the consequence of a series of complicated interactions, and one of the possible measurable factors is the titre of *w*Mel. In the present study, we measured the relative titre and copy number of *wsp* gene of *w*Mel in the new host during different developmental stages and different generations after transinfection by using qPCR. The results showed that *w*Mel could be transmitted from generation to generation, but the relative titre of *w*Mel changed drastically between different developmental stages and different generations. Specifically, the titre of *w*Mel reached a low level during different developmental stages in the 2^nd^ generation (G_2_), and then climbed to the peak in the 6^th^ generation (G_6_), from where the titre of *w*Mel dropped again to a very low level in the 7^th^ and 8^th^ generations, followed by a weak rebound (Fig. 3A). The titre of *w*Mel was persistently low in male adults, but at a higher level and with a more fluctuating mode in female adults; the titre of *w*Mel was higher during nymphal stage than adult stage. Correspondingly, the copy number of *w*Mel in nymphs was also higher than that in adults, and male adults had lower copy number of *w*Mel than female adults (Fig. 3B). The copy number of *w*Mel in female adults climbed up from the low point at G_2_, reaching to a peak at G_6_, and then declined. Similar trend occurred in male adults and nymphs. The highest copy number of *w*Mel in nymphs (52.6 × 10^6^ copies/μl at G_6_) was nearly five times that in male adults (11.1 × 10^6^ copies/μl at G_6_). After G_6_, the copy numbers of *w*Mel in the host gradually decreased and maintained at a comparatively low level.

### Correlation between the titre and transmission rate of *w*Mel

Linear regression analyses showed that *w*Mel titre was not significantly correlated with its transmission rate over a period of 12 generations (*R*^*2*^ = 0.10–0.35) (Table 2). We also analysed the correlation between the copy number of *wsp* gene and the titre of *w*Mel, showing that the copy number of *wsp* gene was positively correlated with the titre (*R*^*2*^ = 0.60–0.80) in adults and nymphs (Supplementary Fig. S3). These data indicate that the relative titre can largely represent the copy number of *w*Mel, and the transmission rate of *w*Mel was most probably not determined by its titre.

## Discussion

The *w*Mel *Wolbachia* strain was known for its CI induction and blocking of dengue transmission after transferred from its original host *D. melanogaster* into the dipteran *Aedes* host^28^. In this study, we showed that the *w*Mel strain could establish and induce a strong CI phenotype in a distantly related insect host of agricultural importance after transferred into *B. tabaci* through microinjection. FISH analysis demonstrated that *w*Mel was localised inside the bacteriocytes along with the primary endosymbiont *Portiera* and could be transmitted into the offspring through the egg; qPCR and transmission efficiency analyses indicated that *w*Mel could comparatively rapidly be adapted to a phylogenetically distantly related host and establish in the new host after only several generations. Moreover, our study revealed that the relative titre and transmission rate of *w*Mel shared a similar fluctuating mode during different developmental stages over more than 10 generations, but the transmission rate is probably not determined by the titre.

FISH is a useful technique for identification and localisation of bacterial endosymbionts in insect species as it can not only indicate whether the symbiont is present but also where it is. Our FISH results were significant because they provided hard evidence for the presence of *w*Mel strain in the new host at various developmental stages (Supplementary Fig. S2) and also for the vertical maternal transmission of *w*Mel strain through eggs between generations. Our finding that *w*Mel was located with the primary symbiont *Portiera* inside the bacteriocyte is consistent with what has been reported previously^43^. The *w*Mel strain and other two *Wolbachia* strains artificially transfected in our previous studies were all found to share the bacteriocyte with *Portiera* in *B. tabaci*^11^,^20^, which may be an efficient mechanism for vertical transmission of these maternally inherited endosymbionts. Nevertheless, cohabitation of different symbionts (exotic and native; secondary and primary) also enhances their interactions that can significantly influence the dynamics of their co-existence.

The *w*Mel strain has previously been shown to induce a strong CI phenotype in the dipteran *Aedes* mosquito after transinfection^33^, but it is still unclear whether *w*Mel can establish and cause CI in a more distantly related host insect. Here we showed that the *w*Mel strain could induce nearly complete CI between *w*Mel-transfected male and wild-type female whiteflies. The same *Wolbachia* strain can induce totally different reproductive phenotypes in different hosts. For instance, the *w*CauA strain from the almond moth, *Cadracautella*, induced CI in *C. cautella* but male killing in the Mediterranean flour moth, *Ephestia kuehniella*^44^. Our data combined with previous reports indicated that *w*Mel could induce a strong CI phenotype not only in its native host and other dipteran hosts but also the non-dipteran host insect without a significant host effect. Interestingly, a low-level sex-biased ratio (68.13% male offspring) was also observed in the reciprocal crossing between *w*Mel-transfected female and wild-type male whiteflies (WT♂ × TI♀). Nevertheless, data analysis revealed that the difference between the observed and expected (1:1) numbers of male offspring is not significant, and therefore the partial CI in the reciprocal direction (WT♂ × TI♀) is not statistically supported in the present study. Theoretically, sex bias in the reciprocal direction should not have taken place if the wild-type male is not infected with a native *Wolbachia* strain (as shown in FISH analysis; Fig. 1a–d); however, bidirectional CI was observed in two of our previous studies using traninsfected *B. tabaci*^11^,^20^. One possible explanation for this may be that the wild-type *B. tabaci* is likely infected with a low-titre *Wolbachia* strain. The presence of *Wolbachia* infection in natural *B. tabaci* populations was supported by FISH analysis showing a very weak *Wolbachia*-specific signal in wild-type *B. tabaci*^20^ and CI induction between wild-type *B. tabaci* males and antibiotic-treated females (*Wolbachia*^−^)^40^. In this study, no native *Wolbachia* strain was detected in the wild-type *B. tabaci* in both FISH and qPCR analyses, and thus no statistically significant bidirectional CI has been induced.

The CI level or strength of reproductive incompatibility, fitness costs associated with *Wolbachia* infection, and transmission rate from mother to offspring are among the major determinants for the rate and extent of spread of a *Wolbachia* strain in a target population^45^. Our studies showed that *w*Mel could induce a strong CI in the recipient host without imposing a significant cost on host fecundity. Furthermore, the transmission rate of *w*Mel returned to a relatively stable level (>50%) after a drastic fluctuation within less than 10 generations. These experimental results suggest that *B. tabaci* is a highly permissive host for exotic *Wolbachia* strains and CI expression as this insect pest was also shown to easily accept other two artificially transfected *Wolbachia* strains from the distantly related hosts *Scleroderma guani (w*SguBJ) and *Corcyra cephalonica (w*Ccep)^11^,^20^. All arthropods are not permissive for exotic *Wolbachia* strains. For example, experimental interspecific transfer of *Wolbachia* in terrestrial isopods indicated that isopod *Wolbachia* were highly adapted to their hosts and could hardly establish in another host species^46^. A highly permissive host provides us more opportunities to regulate its population by CI-based strategy. Here, *w*Mel-transinfected male whiteflies can induce a nearly complete CI in *B. tabaci* populations, producing almost all male offspring, which seriously distorts the sex ratio of the host population and leads to suppression of the target population.

The titre or infection density of an endosymbiont is among the major parameters to understand the dynamics of its establishment process in the recipient host after transinfection. The titre of a *Wolbachia* strain represents the consequence from the interaction between *Wolbachia* and its host during the infection process. Our qPCR results indicated that, during the initial period of infection (G_0_-G_3_), *w*Mel seemed to be poorly adapted to the internal environment of the host, leading to a relatively low titre of *w*Mel. As infection progressed, *w*Mel became better adapted to the new host, which promoted the titre of *w*Mel to a peak level at G_6_. However, the titre of *w*Mel dropped dramatically after G_6_, which might be caused by an induced “counter-attack” from the host. Different from other report^47^, our study showed that the nymphs persistently hosted higher titre of *w*Mel than adults, indicating a complex mechanism regulating the infection progress of an exotic *Wolbachia* invader in the recipient host over different developmental stages. From G_10_, the titre of *w*Mel began to recover, but it remained at a low level in adults, particularly in male adults, indicating that a weak equilibrium has been reached between the host and *w*Mel for some unknown mechanism. Possible reasons include regulation by the host immune system and symbiont-symbiont interactions^48^,^49^. Identification of the specific factors (molecules) that determine the titre of *Wolbachia* in the host during different developmental stages would help us understand the precise molecular mechanisms underlying the endosymbiont-host interaction. Monitoring the infection dynamics of a *Wolbachia* strain in the target host is relevant to the *Wolbachia*-based strategy for biological control of pest insects. For one thing, the infection density can remarkably affect CI level^50^; for another, a potential *Wolbachia* strain as a biocontrol agent should be highly invasive with a persistent infection and transmission capability. Our correlation analysis suggested that the transmission rate of *w*Mel was not determined by its titre, though they shared a similar dynamic trend over a period of 12 generations, indicating that the transmission mechanism of transfected *Wolbachia* might be much more complicated than expected. For further studies, we will determine the correlation between titre and CI level by conducting more crossing experiments by using transinfected whiteflies from different generations with different *Wolbachia* titres.

In conclusion, the *w*Mel strain isolated from *D. melanogaster* could be transmitted and established in the whitefly *B. tabaci*, a distantly related insect host, in a relatively short period of time. This *Wolbachia* strain could induce a strong CI without imposing a significant cost on host fecundity, suggesting a potential for its use for biological control of *B. tabaci*, a worldwide agricultural insect pest.

## Methods

### Insect rearing

The *w*Mel *Wolbachia* strain for transinfection was isolated from the fruit fly *D. melanogaster* in China Agricultural University, Beijing, China in 2014. The fruit flies were maintained on Maize-Agarose-Yeast culture medium at 25 °C, 65% relative humidity (RH) in a climate incubator under a 12-h light-dark cycle. The recipient host insect *B. tabaci* was maintained on the cotton plants (L14:D10 at 28 °C and 60–80% RH).

### Isolation and MLST typing of *w*Mel

Genomic DNA was extracted from individual insects by using the potassium acetate method as previously described^40^. The purified DNA was used as the template for PCR amplification of the six *Wolbachia* genes (*gatB, coxA, hcpA, ftsZ, fbpA*, and *wsp*) with the specific primers reported in Baldo *et al*.^41^ (Table 3); the PCR products were purified and subcloned for sequencing, and the sequences were then used for MLST typing according to the protocol described in the MLST database (http://pubmlst.org/*Wolbachia*/). We also used the previously reported primers^32^ targeting IS5-WD0516/7 (F: 5′-CCAT CAAGGTCTCTTTCA; R: 5′-TGCAAGGAAAACTAAACCAG; expected size: 2488 bp) and IS5-WD1310 (F: 5′-AGGAGAACTGGTCTACGC; R: 5′-TGTTGCTGAGCTTTG CT; expected size: 745 bp) to determine the sub-strain status of the strain isolated in our study.

### Transinfection by microinjection

*Wolbachia* was purified from 10 fruit flies by using the Percoll density-gradient centrifugation method^11^. Microinjection was performed on the 4^th^ instar nymphs (pseudopupae). The amount of *Wolbachia* for each injection (Nanoliter 2000, World Precision Instruments, USA) was 46 nL in SPG buffer (220 mM sucrose, 4 mM KH_2_PO_4_, 9 mM Na_2_HPO_4_, 5 mM L-glutamate, pH7.4) with a glass needle (Φ0.3 mm, CFT-8201, Jiangsu Rich Life Science Instruments Co., Ltd., China). Approximately 80–100 4th-instar whitefly nymphs were injected for each batch. After injection, the nymph in the dish was placed in a climate incubator until adult emergence (L14:D10 at 28 °C and 60–70% RH). A pair of newly emerged ♀/♂ adults (G_0_) was separately maintained on potted cotton plants for establishment of isofemale lines. Five independent single-pairs were constructed, from which three transinfected isofemale lines (G_1_) were selected for further maintenance based on molecular detection of the parental whiteflies (G_0_) (only the parents detected positive for *w*Mel infection were used). The offspring of transfected (TI) whiteflies was detected for the presence of *w*Mel strain by using the primers *wsp*81F/691R targeting the *wsp* gene of *Wolbachia* (Table 3).

### Localisation by FISH

The egg, nymph and adult (male and female) of transfected whiteflies were prepared for FISH analysis and the wild-type (WT) whitefly was used as the control. The methods used were essentially the same as described^20^. Briefly, whitefly samples were fixed in the Carnoy’s fixative overnight. The fixed samples were immersed in 6% H_2_O_2_ for 6 h for decoloration, and then hybridised overnight with the fluorescent probes, BTP1-Cy3 (5′-Cy3-TGTCAGTGTCAGCCCAGAAG-3′), targeting the 16 S rRNA of the primary symbiont *Portiera*, and W2-Cy5 (5′-Cy5-CTTCTGTGAGTACCGTCATTATC-3′) specific to *Wolbachia* 16 S rRNA^43^. Stained samples were viewed under an Olympus FluoViewFV 1000 confocal microscope (Olympus, Japan).

### Reciprocal crossing and CI analysis

Male and female TI and WT whiteflies were selected for crossing experiments. TI whiteflies were taken from the sixth generation (G_6_) when the infection rate was high. Four different crossing experiments were designed: WT♀ × WT♂; WT♀ × TI♂; WT♂ × TI♀, and TI♀ × TI♂. The mating pair was confined in a leaf-clip cage on a cotton plant for 5 days, and then both male and female were removed for molecular detection as described^20^. The positive male or female was designated as TI♂ or TI♀. The eggs laid on cotton plants were placed into climate incubator for further development till adult emergence (L14:D10 and 65% RH at 27 °C). The progenies were collected, and the number of offspring per female and the percentage of males were calculated. CI level was assessed as the proportion of male progenies.

### Maternal transmission rate

Maternal transmission rate was measured as the proportion of infected adult progenies from infected mothers over a total of 12 generations (G_0_ to G_11_). *B. tabaci* is a haplodiploid species, and CI will not increase the proportion of infected offspring because unfertilised eggs will not die but produce male adults. *Wolbachia* infection was detected by using *wsp*-specific primers with total genomic DNA as template^11^. The PCR program was set as follows: 95 °C for 4 min, followed by 2 cycles of touchdown amplification (94 °C for 35 s, 62 °C → 53 °C for 30 s, 72 °C for 30 s), and 20 cycles of 94 °C for 45 s, 42 °C for 45 s, 72 °C for 45 s, and a final extension at 72 °C for 10 min. Ten individuals were detected (*n* = 10) for each generation, with three biological replicates (*N* = 3).

### Titre analysis by qPCR

The relative titre and copy number of *w*Mel in *B. tabaci* at different developmental stages (egg, nymph and adult) were measured by using real-time quantitative PCR over 12 generations after transinfection. The qPCR assay was conducted based on the single-copy gene *wsp* encoding the surface protein of *Wolbachia*. Each DNA sample was extracted from 20 individuals, and the target gene (*wsp*) was amplified, purified, sequenced and then ligated into pGEM-T vector as previously described^11^. The relative titre was measured with *β*-actin as the internal control. The qPCR primers *wsp*Q384/*wsp*Q513 were designed to specifically detect *w*Mel strain (Table 3). The qPCR reactions were performed by using 10 μl of the Platinum SYBR Green qPCR Supermix-UDG (Invitrogen), 0.4 μl of each primer (10 μM), 1.0 μl gDNA and nuclease-free water in a final volume of 20 μl. For absolute qPCR, a standard curve was drawn through five consecutive dilutions with the fusion plasmids (plasmid + insert). The number of *wsp* gene copies (*N*) per microliter was determined by using the protocol as described^51^. To ensure the validity of the data, each measurement was performed in three independent biological replicates and the wild-type whitefly was used as the control. The cycling conditions were: 2 min activation at 95 °C, 40 cycles of 15 s at 95 °C, 30 s at 60 °C. A melting curve was generated under the thermal conditions from 60 °C to 99 °C with a 1-°C rise at each step and a waiting period of 5 s between steps (ABI 7500).

### Data analysis

Statistical differences between groups were analysed by using Student Newman Keuls (SNK) multiple range test of One-way ANOVA at α = 0.01 and 0.05 levels. Error bars in all graphs represent standard error. *Chi*-square test was conducted to analyse the difference in the number of male offspring between the expected 1:1 sex ratio and the crossing results from transinfected and wildtype whiteflies. Linear regression analysis was run on SPSS 20.0 to quantify the relationships between the gene copy number, titre and transmission rate. Transmission rates (%) are transformed by arcsine square root before analysis.

## Additional Information

**How to cite this article**: Zhou, X.-F. and Li, Z.-X. Establishment of the cytoplasmic incompatibility-inducing *Wolbachia* strain *w*Mel in an important agricultural pest insect. *Sci. Rep.* **6**, 39200; doi: 10.1038/srep39200 (2016).

**Publisher's note:** Springer Nature remains neutral with regard to jurisdictional claims in published maps and institutional affiliations.

## Supplementary Material

Supplementary Information

## Figures and Tables

**Table 1:** Crossing between *w*Mel-transinfected (TI) and wild-type (WT) whiteflies.

Cross type (♀ × ♂)	No. of crosses (N)	No. of offspring per female	Percentage of male offspring (%)
WT × WT	11	17.1 ± 0.87 ABab	53.56 ± 2.49 Aa
WT × TI	16	16.1 ± 0.53 Bb	97.46 ± 1.33B
TI × WT	11	17.7 ± 0.84 ABab	68.13 ± 1.24 C
TI × TI	14	19.8 ± 0.97 Aa	58.35 ± 1.89 Aa

The whiteflies are taken from G_6_. The data are mean ± SE. Different letters in the same column indicate significant difference based on SNK test of One-way ANOVA (lowercase letter, *P* < 0.05; uppercase letter, *P* < 0.01).

**Table 2:** Correlation between copy no. of *wsp*, relative titre and transmission rate of the *w*Mel strain in *Bemisia tabaci*.

		Copy no. of *wsp*			Relative titre		
				Female	Male	Nymph	Female	Male	Nymph
**Transmission rate**		0.464	0.475	0.189	0.347	0.123	0.310
**Copy no. of *wsp***	Female				0.705		
	Male					0.618	
	Nymph						0.788

Transmission rates (%) are transformed by arcsine square root before analysis. All data are *R*^*2*^ values based on linear regression analysis.

**Table 3:** The primers used for sequencing, PCR-based detection and real-time qPCR analysis.

Gene name	Primer sequence (5′–>3′)	Fragment size (bp)
*gatB*	F:GAKTTAAAYCGYGCAGGBGTTR:TGGYAAYTCRGGYAAAGATGA	471
*coxA*	F:TTGGRGCRATYAACTTTATAG R:CTAAAGACTTTKACRCCAGT	487
*hcpA*	F:GAAATARCAGTTGCTGCAAA R:GAAAGTYRAGCAAGYTCTG	515
*ftsZ*	F:ATYATGGARCATATAAARGATAG R:TCRAGYAATGGATTRGATAT	524
*fbpA*	F:GCTGCTCCRCTTGGYWTGAT R:CCRCCAGARAAAAYYACTATTC	509
*wsp*	81 F:TGGTCCAATAAGTGATGAAGAAAC 691 R:AAAAATTAAACGCTACTCCA	632
*β-actin*	F:CTTCCAGCCATCCTTCTTG R:CGGTGATTTCCTTCTGCATT	130
*wspQ384 wspQ513*	F:TGGAACCCGCTGTGAATGAT R:GCACCATAAGAACCGAAATAACG	130
