# Supplementary material for: Establishment of the cytoplasmic incompatibility-inducing Wolbachia strain wMel in an important agricultural pest insect
Source: Sci Rep. 2016 Dec 16;6:39200. doi: 10.1038/srep39200 (PMC5159839; doi:10.1038/srep39200)
Supplement: Supplementary Information [file srep39200-s1.pdf]

Establishment of the cytoplasmic incompatibility-inducing *Wolbachia* strain *w*Mel in an important agricultural pest insect

Xiao-Fei Zhou, Zheng-Xi Li*

Department of Entomology, China Agricultural University, 2 Yuanmingyuan West Road, Beijing 100193, China

*Author for correspondence: Dr. Zheng-Xi Li, Department of Entomology, China Agricultural University, 2 Yuanmingyuan West Road, Beijing 100193, China. Tel: +86 10 62732539; Fax: +86 10 62732539; E-mail: zxli@cau.edu.cn.

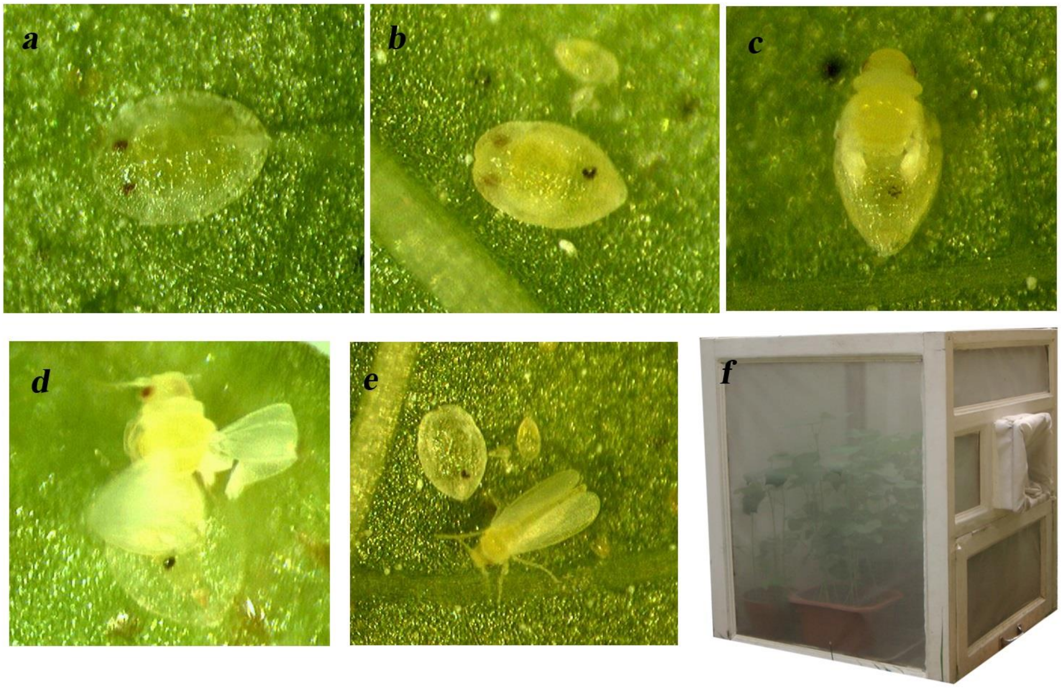

Supplementary Figure S1. Microinjection of *w*Mel strain and establishment of isofemale lines. a, the 4th instar nymph (pseudopupa); b, 24h after injection; c, 48h after injection; d, 72h after injection (eclosion); e, G_0 adult; f, caged population of *w*Mel-transfected *B. tabaci*.

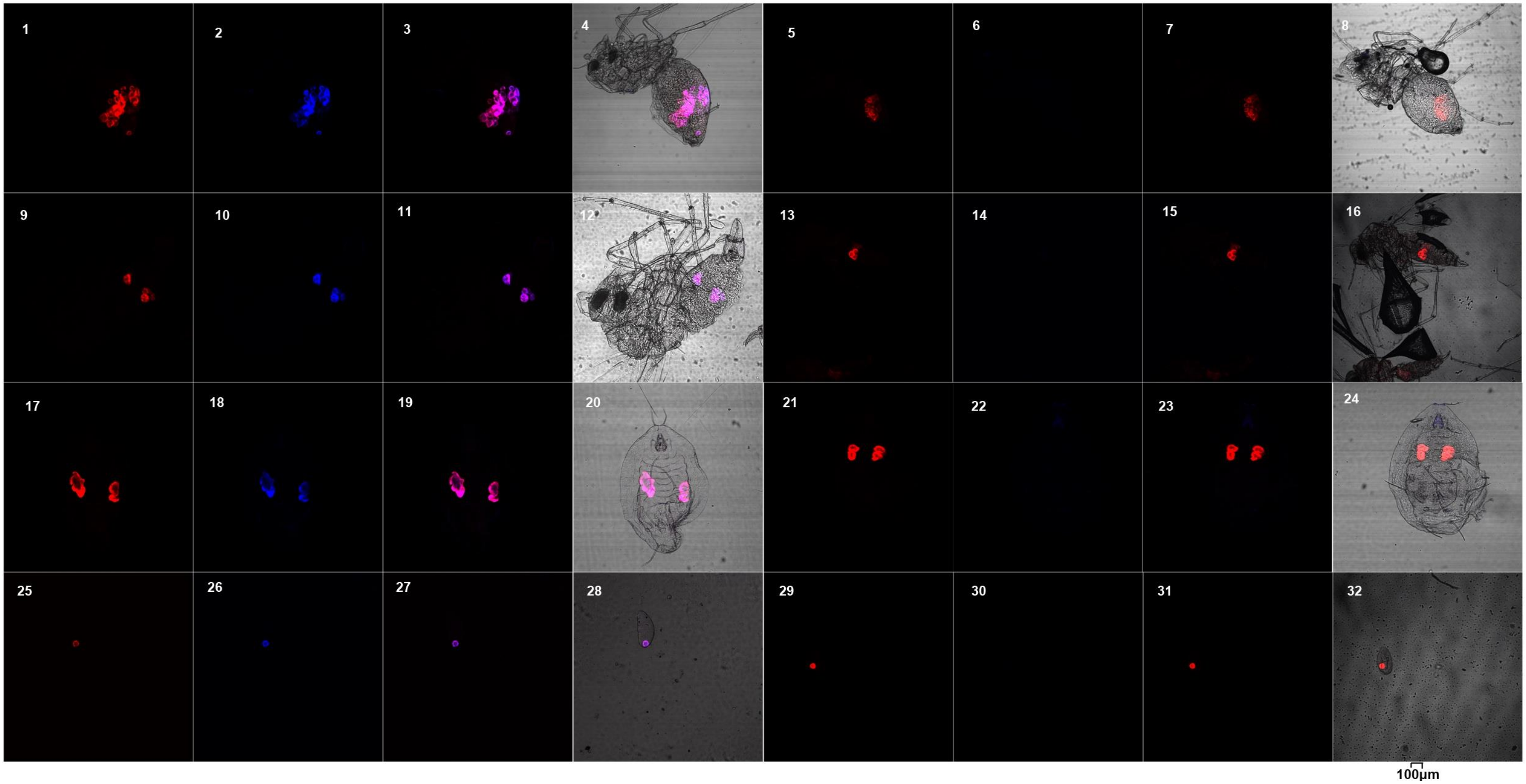

Supplementary Figure S2. FISH analysis of *w*Mel-transfected and wild-type *B. tabaci* at different developmental stages by using *Portiera*-specific probe (red) and *Wolbachia*-specific probe (blue). 1-4: Transfected female adult; 5-8: Wild-type female adult; 9-12: Transfected male adult; 13-16: Wild-type male adult; 17-20: Transfected nymph; 21-24: Wild-type nymph; 25-28: Transfected egg;

29-32: Wild-type egg. 1, 5, 9, 13, 17, 21, 25 and 29, *Portiera* channel only; 2, 6, 10, 14, 18, 22, 26 and 30, *Wolbachia* channel only; 3, 7, 11, 15, 19, 23, 27 and 31, merged images showing overlap of *Wolbachia* and *Portiera* channels in dark field; 4, 8, 12, 16, 20, 24, 28 and 32, merged images showing overlap of *Wolbachia* and *Portiera* channels in bright field.

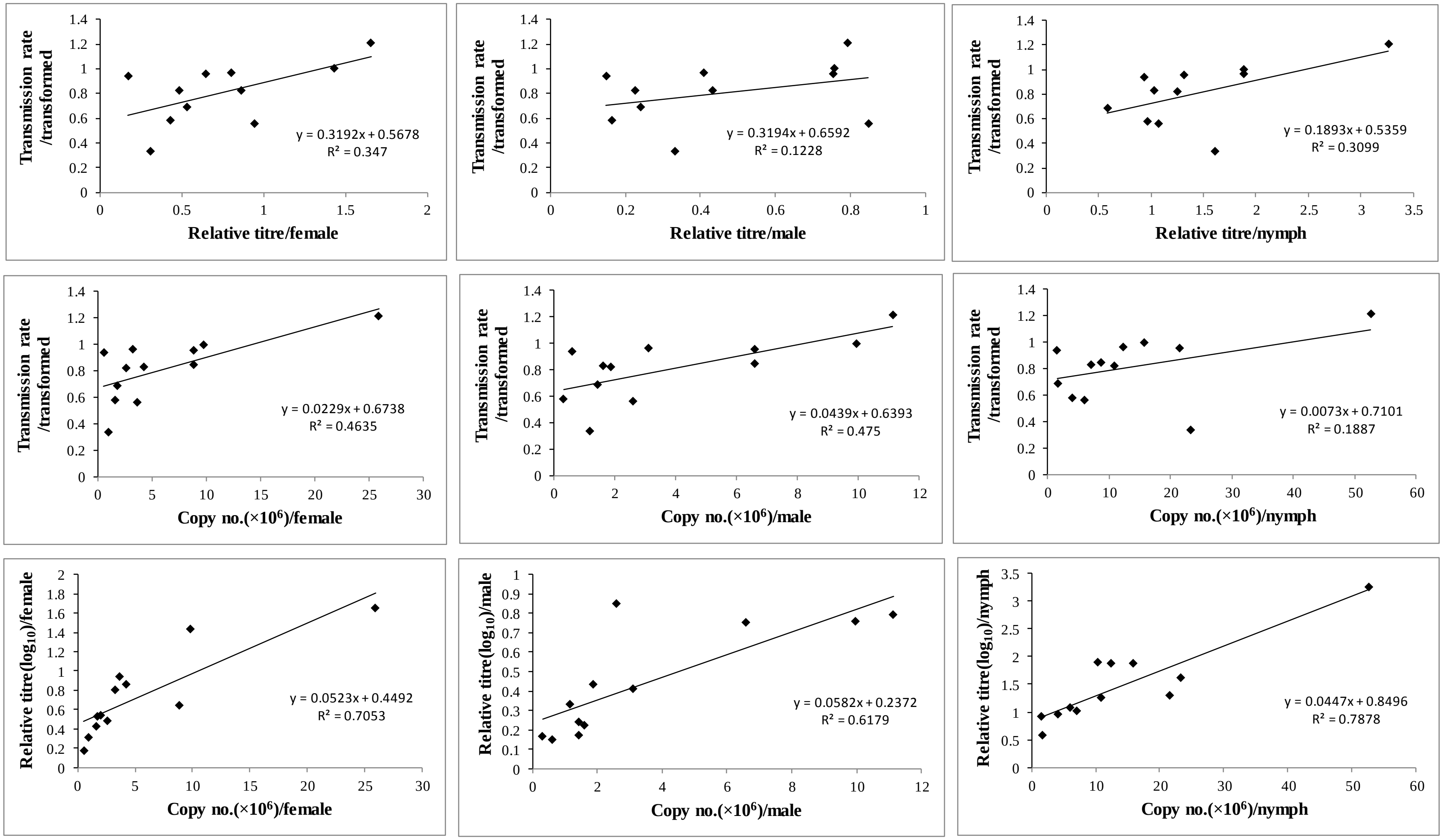

Supplementary Figure S3. Linear regression analysis of the correlation between copy number of *wsp* gene, relative titre and transmission rate of *w*Mel. Transmission rates (%) are transformed by arcsine square root before analysis. a-c, correlation between

relative titre of *w*Mel in female adult (a), male adult (b) and nymph (c) and transmission rate; d-f, correlation between copy number of *wsp* gene of *w*Mel in female adult (d), male adult (e) and nymph (f) and transmission rate; g-i, correlation between copy number of *wsp* gene and relative titre of *w*Mel in female adult (g), male adult (h) and nymph (i).

Supplementary Table S1. MLST and *wsp* sequences and PubMLST query results

Gene name	Fragment size (bp)	Allele sequence (5'-->3')[a]	Allelic type[b]
hcpA	515	GAAATAGCAGTTGCTGCAAAGCAAGGGCTGCCCGATCCCGAACTCAACCCGCGCCTTCGCTCTGCTATATTTGC<br>TGCACGCAAGGAAAATCTACCAAAAGATAAAATAGAAACAGCAATAAAAAATGCAACTGGTAACGTTGCTGGAG<br>AAAATTACGAGGAAATCCAATATGAAGGTCATGGGCCTTCTGGCACTGCACTCATTGTCCATGTTTTGACTAAT<br>AATCGCAACCGAACTGCTTCTGAGGTACGTTATATATTTTCTCGCAAGGGTGGAAACTTGGGAGAAACAGGAAG<br>TGTTAGTTACCTTTTCGATCATGTAGGCTTAATCGTCTATAAAGCAGAGGGTGTGAATTTTGATGATTTATTCA<br>GTCATGGAATCGAATTAGAAGTATTGAATGTTGAGGAAAATGACAAAGAAGGATTACACGTTATAACTTGTGAA<br>ATAAAAGATTTTGGTAAAGTACGCGATGCCTTTTATGCAAAATTCGGAGAACCAGAACTTGCTCAACTTTC	hcpA:1
fbpA	509	GCTGCTCCGCTTGGCATGATTGAAGCTGGTGCTGCAACTTATGCTGGAATGCTCCCACTTATTTTGAAACTTAA<br>TAGTTCCAACTCTTTACATTCAAAGGATCTAACCTCTGATCAGGCAATAACCTCTTCTGTGAAAGATGCGCTGC<br>GTTTGGGATGCTTAGCTGTCGGATTTACTATATATCCTGGTTCTGCTAAGTGTTTCGATATGATGGAGGAAGCC<br>CGTGGAATCATAGCTGAAGCCAAATCTTATGGACTTGCAGTAGTGCTATGGTCTTATCCACGCGGTGAAGGGAT<br>TTCCAAAGAAGGTGAAACAGCAGTTGATGTTATTGCCTATGCTGCGCACATGGCAGCTTTGCTTGGCGCTAATA<br>TAATAAAAGTAAAACTTCCAACTAAATATTTGGAAAGGGAGAAAATAGAAACAGAAAATATTGAATCATTATCT<br>AAAAGAATTGAATATGTTAAAAGGTCTTGTTTTGCAGGGAAAAGAATAGTGATTTTCTCTGGCGG	fbpA:1
gatB	471	GATTTAAACCGTGCAGGGGTTGCTTTAATGGAAATTGTTTCAGAACCAGATCTCCGTTCATCTGCGGAAGCTGC<br>AGAATGCATGAAAAAATTGAGGCAGATTTTGCGTTACATTGGTTCGTGTGATGGTGATATGGAAAAGGGATCAC<br>TTCGTTGTGATGCAAATGTTTCTGTCCGCCTAAAAGGCAGTAGTACATTTGGCACTCGTTGTGAAATAAAAA<br>ATCTGAACTCGATACGTTATATTGTGCAAGCTATAGACTATGAAATACAAAGACAAATTGAAATTTTAGAAAGT<br>GGAGAAGAAATAAGTCAAGATACCTTATTGTTTGACGTTGCTTCGGGAAAAACAAAAGTGATGAGAAGCAAAGA<br>GAATGCAAGCGATTATAGATACTTCCCTGAGCCTGATTTATTACCTGTTGAGGTAAGCCAGGATAAAATTGA<br>TTTAATTCAATCATCTTTGCCTGAGTTGCCA	gatB:1
ftsz	524	ATTATGGAGCATATAAAGGATAGTCATATGCTTTTCATCACAGCAGGAATGGGCGGTGGTACTGGAACCGGTGC<br>AGCACCGGTAATTGCAAAAGCAGCCAGAGAAGCAAGAGCCGCAGTTAAGGATAGAGCGCCAAAAGAAAAAAAGA<br>TATTGACTGTTGGAGTTGTAACTAAACCGTTCGGTTTTGAAGGTGTGCGCCGTATGCGCATTGCAGAGCTTGGA<br>CTTGAAGAACTGCAAAAATACGTGGATACACTTATTGTCATTCCAAATCAGAATTTATTTAGAATTGCAAATGA<br>AAAAACTACATTTTCTGATGCATTTAAACTTGCTGATAATGTTCTGCACATTGGCATCAGAGGAGTAACTGACT<br>TGATGGTCATGCCAGGGCTTATTAATCTTGACTTCGCTGATATAGAAACAGTAATGAGCGAGATGGGCAAAGCG<br>ATGATCGGCACCGGAGAGGCAGAAGGAGAAGATAGAGCAATTAGTGCTGCAGAGGCTGCAATATCCAATCCATT<br>GCTCGA	ftsz:1

coxA	487	TTGGGGCAATCAACTTTATAGTTACTATATTTAACATGCGCACAAAAGGAATGTCATTAACTAAGATGCCACTG<br>TTTGTTTGGTCTGTCTTGCTAACAGCATTTATGTTGATTGTTGCCTTACCAGTGCTTGCCGGTGCTATAACTAT<br>GCTTCTTACTGATCGCAATATTGGTACTTCCTTTTTTGATCCTGCCGGTGGCGGCGATCCTGTGTTATTTCAAC<br>ATCTATTTTGGTTTTTTGGTCATCCAGAAGTTTACGTAATTATTTTTCCTGCATTTGGCATCATAAGTCAGGTT<br>GTATCAACTTTTTCTCACAGACCTGTATTTGGTTACATAGGGATGGTTTATGCAATGATAGGTATAGCAGTATT<br>TGGCTTTATGGTTTGGGCTCACCATATGTTCACTGTTGGGCTTAGTGCTGACGCTGCTGCATTTTTTAGCACTA<br>CCACAATTTTTATCGGTGTTATAACTGGCGTCAAAGTCTTTAG	coxA:1
wsp	595	TGGTCCAATAAGTGATGAAGAAACTAGCTACTACGTTCGTTTGCAATACAACGGTGAATTTTTACCTCTTTTCA<br>CAAAAGTTGATGGTATTACCTATAAGAAAGACAAGAGTGATTACAGTCCATTAAAACCATCTTTTATAGCTGGT<br>GGTGGTGCATTTGGTTACAAAATGGACGACATCAGGGTTGATGTTGAAGGAGTTTATTCATACCTAAACAAAAA<br>TGATGTTAAAGATGTAACATTTGACCCAGCAAATACTATTGCAGACAGTGTAACAGCAATTTCAGGATTAGTGA<br>ACGTGTATTACGATATAGCAATTGAAGATATGCCTATCACTCCATACATTGGTGTTGGTGTTGGTGCAGCGTAT<br>ATTAGCACTCCTTTGGAACCCGCTGTGAATGATCAAAAAAGTAAATTTGGTTTTGCTGGTCAAGTAAAAGCTGG<br>TGTTAGTTATGATGTAACTCCAGAAGTCAAACTTTATGCTGGAGCTCGTTATTTCGGTTCTTATGGTGCTAATT<br>TTGATGGAAAAAAAAACAGATCCTAAAAATTCAACCGGACAGGCTGCTGATGCAGGCGCATACAAAGTTCTTTAC<br>AGCASTGTTGGTGCAGAAGCTGGAGTAGCGTTTAATTTTT	HVR1:1<br>HVR2:12<br>HVR3:21<br>HVR4:24

[a] The underlined bases are sequences of sense and antisense primers.

[b] The allelic profile for gatB:1, coxA:1, hcpA:1, ftsZ:1 and fbpA:1 is corresponding to ST-1; the HVR profile for HVR1:1, HVR2:12, HVR3:21 and HVR4:24 is corresponding to ST-31.
